# A study on the factors causing bottleneck problems in the manufacturing industry using principal component analysis

**DOI:** 10.1016/j.heliyon.2021.e07020

**Published:** 2021-05-20

**Authors:** S.O. Ongbali, S.A. Afolalu, S.A. Oyedepo, A.K. Aworinde, M.A. Fajobi

**Affiliations:** Department Mechanical Engineering, Covenant University, Ota, Ogun State, Nigeria

**Keywords:** Bottleneck problem, Variable, Factors, Analysis, Manufacturing system, Industry

## Abstract

There are a myriad of bottleneck variables that constrict the overall manufacturing capacity and make improvement decisions complex. Consequently, this study aims to identify and analyse the copious variables to pinpoint the key variable factors that influence and turn the manufacturing elements into bottleneck problem to prioritize process improvement effort. The study is limited to identifying and analysing the numerous bottleneck variables to gain insight into how much each of the variables influences the process output via the manufacturing elements. The 76-bottleneck variables abstracted from the literature were used to craft a structured questionnaire that were administered to respondents in the manufacturing industry whose size was determined at a 95% confidence level and 5% error margin respectively. The 95% confidence level is chosen to ensure adequate representation of the population and to validate the data for the study. The respondents' scores were collated into (*m x n*) data matrix which served as input variable into the factor analysis model. StatistiXL software was then employed to evaluate the data matrix. The trivial variables were discarded and 19 factors with eigenvalues (λ ˃ 1) were extracted and creatively labelled for interpretation. The result established that the “Process capability index” is the principal bottleneck factor that loaded 25% of the variables studied. The principal variables in the cluster include Equipment failure = -0.832, Operations = -0.780, Material unavailability = -0.811, and Market demand = -0.739 among others. Similarly, Manufacturing process restraint, Resources, Weather, Communication, Logistics, and Line dedication are other key factors by the magnitude of their respective variables’ factor loadings such as Random event = 0.812, Raw materials flow = -0.834, Process technology = 0.878, and Random environmental factors among other variables. Although bottleneck problems vary from one manufacturing system to another, the problems identified and the solutions presented in this study are generic and the improvement effort should focus on addressing the principal variables while not neglecting the middling and weakling variables.

## 1 Introduction

Bottleneck problems from time-to-time slow-down and occasionally stop the entire manufacturing processes thereby limiting the manufacturing capacity (Lenort and Samolejová, 2007). asserted that all manufacturing systems are constrained by one or more bottleneck problems signifying that irrespective of how well a manufacturing system is designed it cannot be bottleneck-free including the digital manufacturing system or the industry 4.0 revolution. However, a manufacturing asset becomes a constraint on account of variable factors that influence the performance of the asset. Variables constituting bottleneck problems in the manufacturing environment are enormous and therefore compound the improvement decisions occasioned by a lack of direction from where to start addressing the problem. Hence, the challenge is how to identify the principal variables among the numerous variables causing the problem to sharpen process improvement focus and minimize manufacturing losses (Wang et al., 2005). and (Leporis and Zedenka, 2010) argued that any element of production can turn into a bottleneck by causing congestion, slow down or stop the manufacturing process. According to (Lawrence and Buss, 1995), bottlenecks are inevitable when there are differences in job arrival and processing rates. The consequence of bottleneck problem on manufacturing system is loss of economic value because it defines the volume of the manufacturing outputs (França et al., 1997) (Yano and Lee, 1995) (Bassok and Akella, 1991) (Lenort and Samolejová, 2007) (Roser et al., 2001) (Ongbali Samson et al., 2018).

Improving the bottleneck problems will enhance the overall manufacturing system (Roser et al., 2002, 2002b). However, process improvement can only be achieved through bottleneck detection and identification (Kuo et al., 1996) (Chiang et al., 2000). For example (Roser et al., 2002, 2002b), formulated a model to detect and identify the bottleneck problem in a manufacturing system based on the active duration for which a machine can run without interruption. This approach does not take into account identification of the variable factor makes the manufacturing asset to become a bottleneck in the manufacturing process. In our opinion, a system bottleneck can be estimated as,

(1) $$System\;Bottleneck\;=\;\frac{Actual\;output\;capacity}{Install\;capacity}\;x\;100\%$$

The study focuses on holistic identification and analysis of a myriad of variables causing bottleneck problems in the manufacturing setting to gain insight into the key variables influencing the overall performance of the manufacturing system to prioritize improvement effort. Thus, the resources reviewed to obtain the bottleneck variables have no specific period because bottleneck variables remain constant irrespective of when they are discovered. Table 1 depict references of manufacturing bottleneck variables.

**Table 1:** References of manufacturing bottleneck variables.

Bottleneck variable	Reference
Machine breakdown	(Wazed et al., 2010); (Nakata et al., 1999); (Chiang et al., 2001); (Riezebos et al., 2003); (Roser et al., 2002, 2002b); (Leporis and Zedenka, 2010); (Liu et al., 2004); (Sui-Hua, 2005); (Hwang and Singh, 1998); (Kuo et al., 1996); (Biller et al., 2010); (Chiang et al., 2000); (Chiang et al., 1998); (Yano, 1987); (Identifying Bottleneck Cells with Goal Programming, 2008); (Sengupta et al., 2008); (Roser et al., 2002, 2002b); (Gunasekaran et al., 1998)
Quality variation	(Wazed et al., 2010); (Biller et al., 2010); (Chiang et al., 2000); (Chiang et al., 1998); (Ben-Daya and Rahim, 2003); (Identifying Bottleneck Cells with Goal Programming, 2008)
Process variation	(Wazed et al., 2010); (Jiang et al., 2003); (Sharda and Bury, 2010); (Grosfeld-Nir and Gerchak, 2004); (Biller et al., 2008); (Biller et al., 2010); (Ben-Daya and Rahim, 2003); (Identifying Bottleneck Cells with Goal Programming, 2008); (Gunasekaran et al., 1998)
Inspection error and time	(Wazed et al., 2010); (Darwish and Ben-Daya, 2007); (Ben-Daya and Rahim, 2003); (Identifying Bottleneck Cells with Goal Programming, 2008)
Set-up and change-over time	(França et al., 1997); (Grosfeld-Nir and Ronen, 1993); (Yano, 1987); (Ong et al., 2005); (Luthi, 2002)
Location	(Chen and Ya-Wen, 2008)
Yield uncertainty	(Tang, 2009)
Random variation	(Nakata et al., 1999); (Kozan, 2001); (Roser et al., 2002, 2002b)
Ergonomic hazards	(Adler et al., 1997)
Forecast errors	(Spearman and Zazanis, 1992)
Buffer mechanisms	(Spearman and Zazanis, 1992); (Leporis and Zedenka, 2010); (Biller et al., 2008); (Chiang et al., 1998); (Yano, 1987)
Product structure	(Spearman and Zazanis, 1992)
Facility layout	(Spearman and Zazanis, 1992)
Equipment failure	(Spearman and Zazanis, 1992); (Leporis and Zedenka, 2010); (Lawrence and Buss, 1995)
Inventory policy	(Spearman and Zazanis, 1992); (Cortazar and Schwartz, 1993); (Lawrence and Buss, 1995); (Vickery and Markland, 1985)
Lot-sizing and batching policy	(Spearman and Zazanis, 1992); (Jain et al., 2003); (Liu et al., 2004); (Yano, 1987); (Gunasekaran et al., 1998)
Price volatility	(Cortazar and Schwartz, 1993)
Insufficient Resources	(Ronen and Spector, 1992); (Kozan, 2001); (Wang et al., 2005); (Hwang and Singh, 1998); (Lawrence and Buss, 1995),
Market demand	(Ronen and Spector, 1992); (Lenort and Samolejová, 2007)
Environmental factors	(Yano and Lee, 1995); (Grosfeld-Nir and Gerchak, 2004)
Materials	(Yano and Lee, 1995); (Bassok and Akella, 1991); (Lenort and Samolejová, 2007); (Grosfeld-Nir and Gerchak, 2004)
Information and material flow	(Cipollone and Marchetti, 2001)
Information and time-of-usage	(Arnott et al., 1999)
Random job arrival	(Bassok and Akella, 1991); (Liu et al., 2004); (Lawrence and Buss, 1995); (Bukchin, 1998); (Suresh and Whitt, 1990); (Dai et al., 1994)
Capacity limitation	(Wazed et al., 2010); (Jiang et al., 2003); (Bassok and Akella, 1991); (Lenort and Samolejová, 2007); (Roser et al., 2001); (Wang et al., 2005); (Lima et al., 2008); (Lawrence and Buss, 1995); (Yano, 1987); (Giuliano and Serazzi, 2003); (Luthi, 2002); (Gunasekaran et al., 1998); (Vickery and Markland, 1985); (Yan et al., 2010)
Task time variation	(Kozan, 2001)
Labor and worker flexibility	(Spearman and Zazanis, 1992); (Lenort and Samolejová, 2007); (Leporis and Zedenka, 2010); (Wang et al., 2005); (Liu et al., 2004); (Hwang and Singh, 1998)
Energy	(Lenort and Samolejová, 2007)
Process technology	(Lenort and Samolejová, 2007); (Sui-Hua, 2005); (Berger et al., 1999)
Operation structure	(Lenort and Samolejová, 2007); (Yano, 1987)
Logistics and supply constraint	(Ahn and Kaminsky, 2005); (Cipollone and Marchetti, 2001); (Liu et al., 2004); (Yano, 1987)
Scheduling policies	(Zhang and Wu, 2012)
Production elements	(Wang et al., 2005)
Functions and Departments	(Wang et al., 2005); (Lawrence and Buss, 1995)
Supply and demand variation	(Wang et al., 2005); (Jain et al., 2003)
Changing machine tools	(Wang et al., 2005); (Hwang and Singh, 1998); (Biller et al., 2008)
Excessive capacity utilization	(Sui-Hua, 2005); (Grosfeld-Nir and Gerchak, 2004)
Rework process	(Sui-Hua, 2005); (Yano, 1987); (Identifying Bottleneck Cells with Goal Programming, 2008); (Bowling et al., 2004)
Specialize skills	(Hwang and Singh, 1998)
Machine layout	(Chiang et al., 1998); (Samson et al., 2019)
Preventive maintenance	(Identifying Bottleneck Cells with Goal Programming, 2008)
Workload variability	(Sengupta et al., 2008); (Gunasekaran et al., 1998)
Demand variation	(Roser et al., 2002, 2002b)
Heterogeneity of products	(Gunasekaran et al., 1998)

The foregoing literature appraisal demonstrates that although manufacturing bottleneck problems had been widely studied, the gap remains that there are no studies focusing on a holistic analysis of the numerous bottleneck variables that turn the manufacturing elements into bottleneck problem to highlight the principal variables causing the problem. Hence, the motivation for this study is to address the gap by identifying and analysing the variables to pinpoint the key variables to guide improvement effort for efficient manufacturing systems.

Section 1 illustrates the situation of study and the literature review while section 2 presents the materials and methods. Section 3 presents results and discussion, and finally, section 4 presents a conclusion to the study.

## 2 Materials and methods

A vast literature review in the topic area was carried out to abstract the pertinent bottleneck variables for the study and seventy-six bottleneck variables were identified. The 76-bottleneck variables were used to craft a structured questionnaire using Renish Likert's 5-point attitudinal scale whose dimensions include strongly agree, agree, undecided, disagree, and strongly disagree.

The representative sample size of the manufacturing industry population selected for the study was determined by using Eq. (2) to justify an adequate population size for the study.

(2) $$Sample\;size=\frac{Z_{\frac{{\left(1-\;\alpha \right)}^{2}}{2}}P\left(1-p\right)}{d^{2}}$$

where $Z_{\frac{{\left(1-\;\alpha \right)}^{2}}{2}}$ = Standard normal variate at 5% error; *p* = Expected proportion in the population and *d* = Absolute error or precision (Charan and Biswas, 2013).

The questionnaires were then administered to the respondents in the manufacturing industry.

### 2.1 A brief theoretical framework to eigenvalues

In Principal Component Analysis (PCA), eigenvalues guide the extraction of factors during varimax rotation which explains the relationship among the factors. Eigenvalues measures the variance of the variable accounted for by a factor, whereupon it determines the number of factors to be selected in the analysis. In the application of matrices to solving engineering problems, Eq. (3) does occur.

(3) Ax=λx

$A\;=\;\left(a_{ij}\right)$ is a square matrix of (*m x n*) which in this context is the correlation matrix. A=unknownnx1vector, λ=unknownscalar.

Clearly, x=0is a trivial solution of λ because the factor does not contribute to the explanation of the variance of the variable. However, our interest is the search for “non-trivial” solutions such that x≠ 0 for any value of λcalled – eigenvalues ofA. Whether the solution is trivial or not depends upon the values of λ. Eq. (3) can be expressed as

(4) Ax−λx=0

Introducing a unit matrix to Eq. (4) yields,

(5) (A−λI)x=0

Where I is (*m x n*) identity matrix. Hence, Eq. (5) can further be expressed as,

(6) $$\left|A-\lambda I\right|=\left|\begin{array}{lll} \left(a_{11}-\lambda \right) & a_{12} & \ldots \ldots \ldots \ldots \ldots \ldots \ldots a_{1n}\\ a_{21} & \left(a_{22}-\lambda \right) & \ldots \ldots \ldots \ldots a_{2n}\\ \cdot & \cdot & \cdot \\ \cdot & \;\cdot & \;\cdot \\ \cdot & \;\cdot & \;\cdot \\ a_{In} & a_{In} & \left(a_{In}-\lambda \right) \end{array}\right|=0$$

|A−λI| is the determinant of A and |A−λI|=0is the characteristic equation. Thus, expanding the determinant gives a polynomial degree of n and the solution of the characteristic equation gives the values of λwhich is the eigenvalues ofA. StatistiXL software can be employed to solve Eq. (6) for the eigenvalues to determine the factors that are relevant to the study. The theory presented in the foregoing has provided clarification and insight into the significance of eigenvalues in the Principal Component Analysis.

### 2.2 Data acquisition

Data for the study was obtained by administering a structured questionnaire to the respondents population in the manufacturing industry after determining the sample size by using Eq. (2) at 95% confidence level. The 95% confidence level was chosen to ensure an adequate representative population size and to validate the data used for the study. The respondents’ scores were collated into (mxn)data matrix.

### 2.3 Data analysis

The (mxn) data matrix obtained served as an input variable into the Principal Component Analysis model. StatistiXL software was then employed to evaluate the data matrix and 19 factors with eigenvalues (λ ˃ 1) were extracted and labelled for meaningful interpretations.

**Figure 1:**
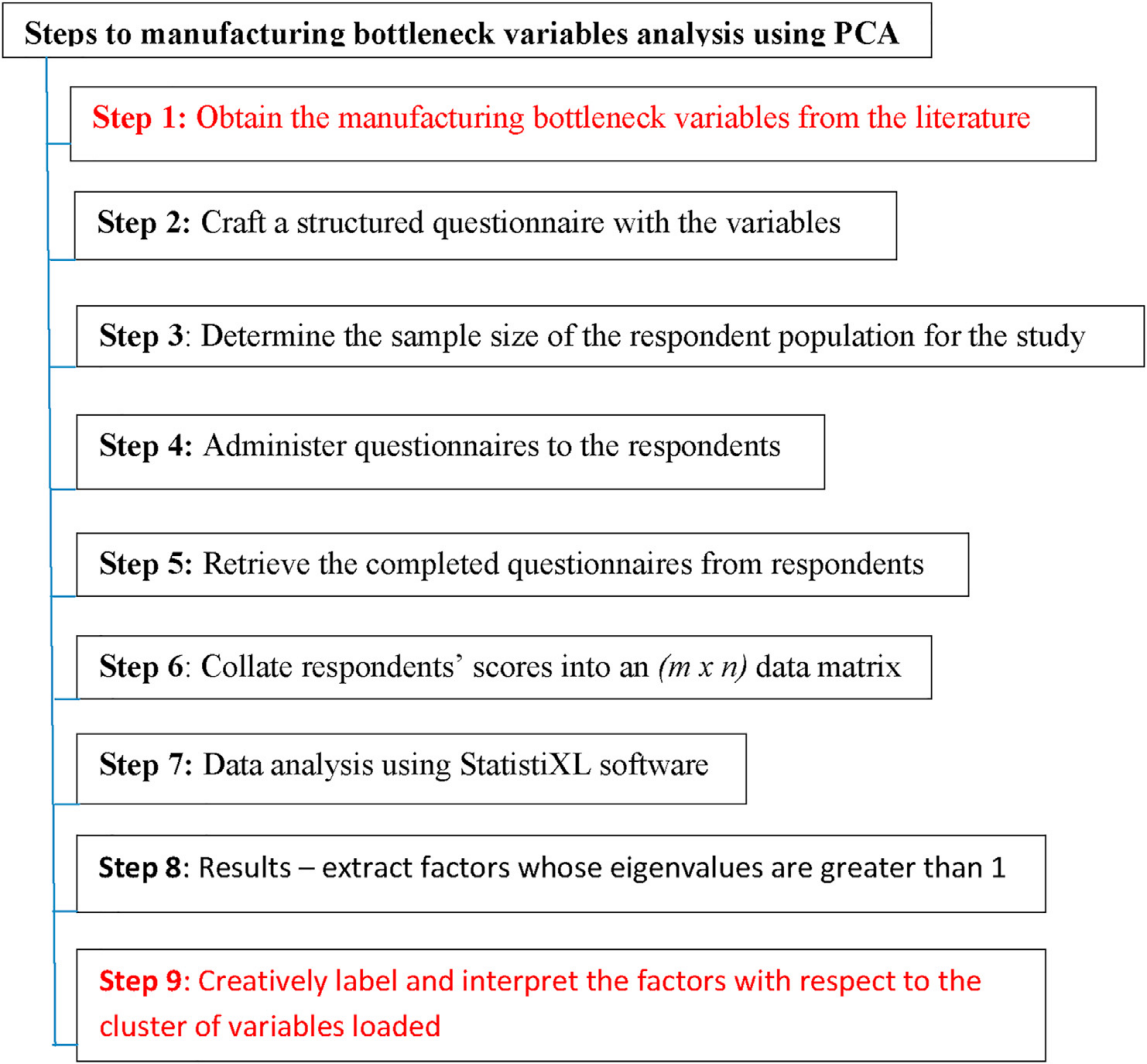
Primary bottleneck variables identification method in the manufacturing setting.

The justification for the selection of the 19 factors having Eigenvalues greater than 1 is because the factors contribute to the explanation of the variance of the variables studied based on their factor loadings (see Tables 3, 4, 5, 6, 7, 8, 9). Figure 1 depicts the step-by-step procedure to bottleneck variable analysis using PCA tool.

**Table 3:** Manufacturing process restraint factor.

F_2_ (8)	Factor loading	Bottleneck variables
1	0.732	Automatically Guided Vehicle (AGV)
5	0.554	Capacity utilization
8	-0.502	Tool changing time
13	-0.537	Complex process adjustment
28	-0.622	Function
54	-0.640	Different models arrival
59	0.812	Random event
75	-0.834	Raw materials flow

**Table 4:** Resources factor.

F_3_ (2)	Factor loading	Bottleneck variables
25	0.729	Facility limitation
57	0.752	Resources

**Table 5:** Communication factor.

F_4_ (2)	Factor Loading	Bottleneck variables
30	0.802	Information and time-of-usage

**Table 6:** Man-machine interface factor.

F_5_ (4)	Factor loading	Bottleneck variables
21	0.711	Ergonomic hazards
24	0.750	Facility layout
48	0.878	Process technology
71	0.565	Worker flexibility

**Table 7:** Logistics factor.

F_6_ (2)	Factor loading	Bottleneck variables
10	0.874	Choice of location
68	0.532	Transportation time

**Table 8:** Weather factor.

F_7_ (1)	Factor loading	Bottleneck variable
55	0.854	Random environmental factor

**Table 9:** Line Dedication factor.

F_8_ (3)	Factor loading	Bottleneck variables
9	0.566	Product mix change
19	0.872	Processing rate

## 3 Results and discussion

**Figure 2:**
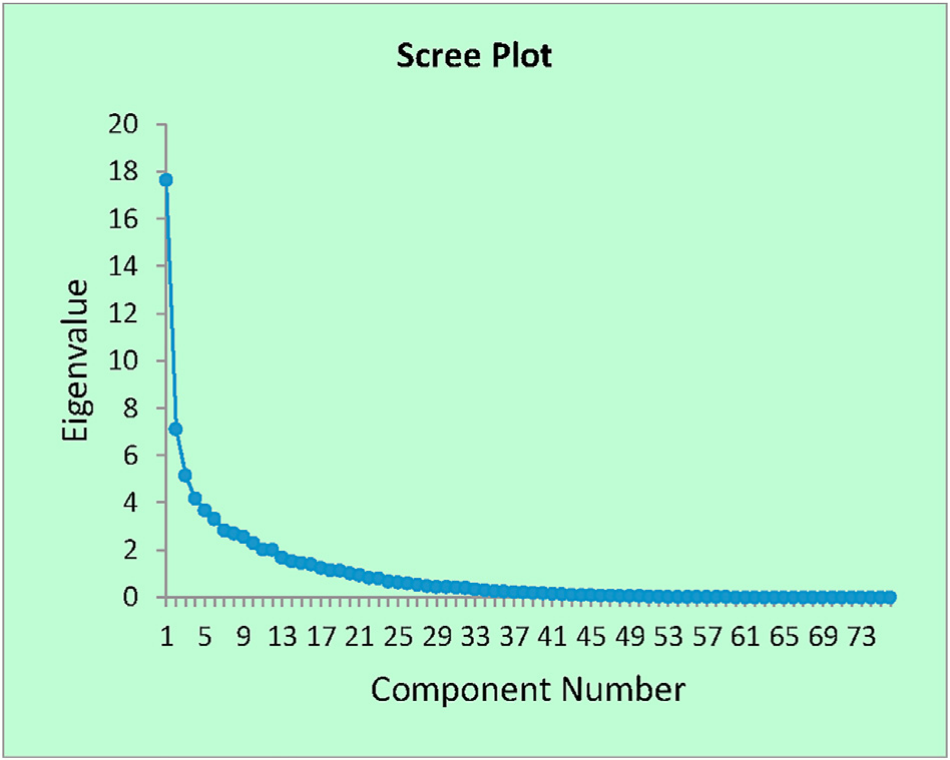
The scree plots of the bottleneck variables.

The nineteen factors extracted from the analysis loaded clusters of variables with varying factor loadings corresponding to the explanation of the amount of variance each variable wield on the manufacturing throughput. Figure 2 depicts the scree plot of the factorial analysis showing the number of factors generated by the analysis. It explains how much variation each principal component exerted in the data examined.

The significance of the scree plot is to determine the numbers of reasonable factors to select for interpretation in the analysis as illustrated in Figure 2. The factors selected from the analysis are labelled and given meaningful interpretation as follow.

Factor (F_1_) is a principal factor that loaded 25% of the variables studied. All the variables have negative factor loadings, thus indicating how negatively the variables affect the manufacturing system or positively otherwise. In particular, the model demonstrated that materials unavailability and equipment failure with factor loadings of -0.811 and -0.832 respectively are the most critical variables. Energy insufficiency is prevalent in the entire economy of the developing countries. Other variables in the group affect the manufacturing process to a degree indicated by the magnitude of their factor loadings. The justification for factor (F_1_) loading 25% of the examined variables shows that it is a critical factor and it contributes largely to the manufacturing problems. Also, the loaded variables share a common correlation and influence the manufacturing system almost in the same manner.

Table 2 is a bipolar factor comprising both positive and negative factor loadings. This platoon bears three critical variables which are Automatically Guided Vehicle (AGV), Random events, and raw materials flow having factor loadings of 0.732, 0.812, and -0.834 respectively. The AGV transports material that cannot be easily handled by humans to various workstations and if it is dysfunctional, it constitutes a bottleneck problem. On the other hand, random events are non-linear activities that have no definite pattern of manifestation and if it is not managed they constitute a serious problem that may stop the entire manufacturing system. Again, raw materials flow is significant in the manufacturing process because raw materials conversion cannot be effective when they are not in the right quantity, at the right place, and at the right time. The rest variables in the platoon are middling by the magnitude of their factor loadings.

**Table 2:** Process capability index factor.

F_1_ (19)	Factor loading	Bottleneck variables
14	-0.635	Non-conforming material
20	-0.686	Energy
22	-0.832	Equipment failure
23	-0.739	Market demand
34	-0.577	Labour insufficiency
37	-0.781	Machine breakdown
38	-0.640	Machine repair
39	-0.709	Tools unavailability
40	-0.618	Materials
41	-0.580	Multi-stage supply logistics
42	-0.780	Operations
43	-0.637	Operators skills
45	-0.765	Preventive maintenance
62	-0.651	Resources shortage
63	-0.533	Scheduling policies
66	-0.769	Supply constraints
67	-0.529	Task time variation
69	-0.811	Material unavailability
70	-0.615	Unscheduled downtime

Resources in this context imply working capital, materials, workforce, equipment, and technology which the manufacturing organization uses to generate revenue. The variables’ factor loadings are substantial ranging from 0.729 to 0.752 thereby displaying how important resources are to the manufacturing process. If resources are in short supply, they constitute a bottleneck. Also, facility limitation constitutes manufacturing bottlenecks to the tune of 0.729.

Information dissemination in manufacturing organizations is quite essential. This includes external and internal communication. The feedback aspect of information is ever vital because it provides an opportunity for redirection and reinforcement for an efficient manufacturing system. The factor loading of 0.802 signifies a huge variance constituted by communication and its significance in the manufacturing system. Effective communication promotes teamwork and leadership in the organization that will eventually translate into profit, product availability, customer satisfaction, and high market share.

Under this factor, process technology wields the highest factor loading of 0.878 which is considered meritorious. Also, facility layout and ergonomic hazard possess substantives factors loadings of 0.750 and 0.711 respectively showing their relative importance in the man-machine interface. The worker flexibility variable under this factor is middling. The four variables loaded by (F_5_) are all human factors engineering issues and they need to be addressed in such a way that the facilities fit workers and hazards in the workplace are identified and curtailed. In so doing, work errors that may lead to accidents that can slow down the job are also minimized or eliminated.

This is a duplex factor showing that the choice of location of a factory is of prime importance by the meritorious factor loading of 0.874. The transportation component wields a middling factor loading of 0.532. This factor suggests that in siting a factory, transportation issues must be taken into consideration. Contiguity of raw materials to production location can help in reducing the cost and time it takes to deliver material to the point-of-use (POS).

Factor (F_7_) is concerned about relative humidity, temperature, dust, and other climatic variables that wield a factor loading of 0.854. The factor suggests that weather variables could affect manufacturing organizations either adversely or favourably and it is therefore important that its effect on the manufacturing process should be of concern.

Factor (F_8_) is another duplex factor consisting of processing rate and product mix change. It has a commendable factor loading of 0.872 for processing rate indicating that the speed with which products are processed and change to another product design in a line would depend on whether a line is dedicated to a product, two or more products in which case, substantial time is spent on retooling and set-up or change-over.

Factors F_9_ to F_19_ loaded variables with middling and weakling factor loadings ranging between 0.316 and 0.491. The followings are the miscellaneous variables and their corresponding factor loadings. Demand variation = -0.453; Buffer capacity = -0.491; Specialize operator skills = -0.474; Worker = -0.457; Forecast errors = -0.538; Inspection errors = 0.398; Process restoration = -0.361; Random yields = 0.440; Random variation = 0.328; Increasing capacity utilization = -0.387; Workload variation = 0.346; Process variation = -0.319 and Job arrival variability = 0.316.

Vast researches in the field of manufacturing bottleneck problems focus on detection and identification of bottleneck elements using different techniques that are well summarized in (Urban and Rogowska, 2020) without taking into account the variable factors that influence the manufacturing element and turn it into bottleneck problem. For instance (Kuo et al., 1996); (Chiang et al., 2000) stated that manufacturing process improvement can only be achieved through bottleneck detection and identification. Similarly (Urban and Rogowska, 2020), claimed that constraint identification in production system is the basis for improvement (Teng et al., 2020). proposed Bottleneck Tree Analysis (BOTA) in conjunction with Green and Lean Index (GLI) for capacity bottleneck identification. Furthermore (Wang et al., 2005), and (Leporis and Zedenka, 2010) argued that any production element can turn into bottleneck by causing congestion and slow down the manufacturing process.

It appeared that previous researches focused only on the identification of production element that constitute bottleneck problem without considering the corresponding variable factors that influence and turn the element into bottleneck problem. Hence, our work focuses on identification and analysis of a myriad of bottleneck variables to pinpoint the primary variables that turn the manufacturing element into bottleneck problem to prioritize process improvement. It is evidence in the literature that identification of the key variable factors that turn the manufacturing element into bottleneck were not previously captured by researchers which is the gap this study has bridged.

## 4 Conclusion

The result of this study established that the “Process capability index” is the principal bottleneck factor that loaded 25% of the variables studied. The principal variables in the cluster include Equipment failure = -0.832, Operations = -0.780, Material unavailability = -0.811, and Market demand = -0.739 among others. Similarly, Manufacturing process restraint, Resources, Weather, Communication, Logistics, and Line dedication are other key factors by the magnitude of their respective variables’ factor loadings. For instance, Random event = 0.812, Raw materials flow = -0.834, Process technology = 0.878, and Random environmental factors among other variables. Although other factors extracted loaded variables with middling and weakling factor loadings, it is recommended that they should not be ignored in manufacturing planning and control.

This paper contribute to knowledge scientifically by pinpointing the primary bottleneck variables among copious variables that influence and turn the manufacturing elements into bottleneck problem which is not previously captured by researchers whom only focused on identification of bottleneck elements in manufacturing system without taking into consideration the corresponding variable factors that influence and turn the elements into bottleneck problem.

The perceived limitation of the study is that the data used for the study was obtained through qualitative means rather than quantitative, therefore, the judgment of the sampled respondents may not reflect 100% the opinion of the unsampled population from the manufacturing industry.

Identification of the manufacturing bottleneck element together with the corresponding variable factors that turn the element into bottleneck prioritizes process improvement. Hence, we recommend future research to consider detection and identification of manufacturing bottleneck element along with the corresponding variable factor that turn the element into bottleneck.

## Declarations

### Author contribution statement

Ongbali S. O., Afolalu S. A. & Oyedepo S. A.: Conceived and designed the analysis; Analyzed and interpreted the data; Contributed analysis tools or data; Wrote the paper.

Aworinde A. K. & Fajobi M. A.: Analyzed and interpreted the data; Contributed analysis tools or data; Wrote the paper.

### Funding statement

This research did not receive any specific grant from funding agencies in the public, commercial, or not-for-profit sectors.

### Data availability statement

Data included in article/supplementary material/referenced in article.

### Declaration of interests statement

The authors declare no conflict of interest.

### Additional information

No additional information is available for this paper.
